# Evaluation of the impact of fluoride in drinking water and tea on the enamel of deciduous and permanent teeth

**DOI:** 10.1186/s12903-023-03267-6

**Published:** 2023-08-13

**Authors:** Asu Çakır, Tuğçe Nur Şahin

**Affiliations:** Department of Pediatric Dentistry, Faculty of Ahmet Keleşoğlu Dentistry, Karamanoğlu Mehmetbey University, Yunus Emre Campus, Karaman, Turkey

**Keywords:** Drinking water, Black tea, Fluoride, Enamel, Turkey

## Abstract

**Background:**

Systemic fluoride intake is predominantly derived from drinking water and tea. It’s been noted that water and tea containing fluoride, within the boundaries set by the World Health Organization, can lessen the prevalence of dental caries. A review of the literature did not uncover any study that has examined the impact of fluoride in bottled drinking water and tea on enamel of deciduous and permanent teeth. Thus, we assessed the effects of fluoride present in seven different brands of bottled water from distinct geographical regions of Turkey, and a type of tea produced and packaged in Turkey, on the enamel of deciduous and permanent teeth.

**Materials and methods:**

Fluoride analysis was performed on drinking water sourced from seven different regions of Turkey and a brand of tea brewed with these waters. The tea was harvested and packaged in Turkey. The analysis was conducted using an ion-selective electrode. In total, 112 tooth enamel samples (56 deciduous molars and 56 permanent molars) were randomly divided into eight distinct groups. These were kept in water for 15 min and tea for 15 min every day for a month. The eighth group was treated with fluoride gel prior to tea and water applications. The amount of fluoride in the tooth enamel structure was evaluated using an SEM EDX device before and after the experiment.

**Results:**

Statistically significant differences were found in fluoride content of enamel between water brands and tooth type (deciduous and permanent teeth). Fluoride levels were higher in the enamel of deciduous teeth than in permanent teeth.

**Conclusion:**

Regular exposure of enamel samples to black tea and water led to an increase in fluoride levels in the enamel; thus, regular consumption of black tea and fluoride water could help reduce the prevalence of dental caries.

## Background

Fluoride use is associated with a reduction in dental caries prevalence and severity. Topically, low fluoride levels in plaque and saliva decrease enamel demineralization and enhance remineralization of demineralized enamel [1, 2]. By interacting with calcium and phosphate ions in the enamel, fluoride precipitates as fluorapatite, lowering enamel solubility and thereby increasing caries resistance [3, 4]. Certain fluoride concentrations have been deemed beneficial for preventing dental caries, with a saliva fluoride concentration of 0.5 ppm (parts per million) or more reportedly initiating caries remineralization [5]. Therefore, low-concentration applications are often recommended for daily use [6–8].

Fluoride can be taken as individual, collective and professional applications. Collective applications are crucial considering that not everyone worldwide can access dental services for topical applications. The World Health Organization (WHO) maintains that while fluoride naturally occurs in both surface and groundwater, it can be added to public water supplies in a controlled manner [9]. Per global standards the recommended fluoride content for drinking water is between 0.5 and 1.5 mg/L [10]. Studies have shown that while fluoride can be found in food, beverages, tea, tobacco, and fish, the levels in drinking water, which is consistently ingested systemically, has the greatest effect [11]. Given that the daily fluoride intake is about 0.05–0.07 mg, if levels exceed 1 mg/L in bottled drinking water, it is mandatory to label it as fluoridated water [12–14]. As fluoride content may vary in spring water, consumers should select water that is suitable for their needs and metabolism by reading the label information. Overdoses of fluoride have been linked to severe dental fluorosis and skeletal fluorosis [15].

Tea is the plant with the highest fluoride content, with each cup of tea containing 0.19–0.31 mg fluoride. In areas with fluoridated water, levels in tea could reach 8–10 mg per day [16, 17]. Turkey reportedly has the highest per capita tea consumption, with an annual intake of 3.5 kg per person [18].

It has been suggested that low fluoride concentrations can remineralize the enamel surface, while higher concentrations can prevent caries by inhibiting protein and bacterial accumulation on the enamel surface [19, 20]. Systemic fluoride application is being increasingly replaced by topical fluorides [21, 22]. Topical fluoride application may be done professionally or individually, and is used to halt, delay, or reverse caries progression [23]. Professional fluoride materials (fluoridated gels, varnishes, intraoral fluoride-releasing products) contain higher fluoride concentrations (5–19 mg) while individual fluoride materials (such as fluoride toothpastes and mouthwashes) contain lower concentrations (0.2–1 mg) [24, 25].

In conclusion, given that dental caries is the most prevalent disease worldwide, studies underscore the importance of systemic fluoride intake, particularly in populations with different sociodemographic levels in countries who lack broad access to dental services. Swallowed fluoride, such as fluoridated water and dietary supplements, may have a direct topical effect on erupted teeth, as well as an effect by increasing salivary and gingival crevicular fluoride levels. In this context, the fact that individuals can derive fluoride at sufficient rates from various foods consumed daily, particularly water and tea, should not be overlooked. However, few studies have examined the effects of fluoride from frequently consumed tea and water to deciduous and permanent teeth. Thus, we evaluated the effect of fluoride from seven different waters sourced from seven distinct geographical regions of Turkey, along with a brand of tea brewed with these waters, on enamel of deciduous and permanent teeth.

## Materials and methods

### Study design and setting

This in vitro study evaluated the impact of different brands of bottled drinking water originating from seven distinct regions of Turkey, along with tea brewed with these waters, on tooth enamel.

### Sample source

The study sample included permanent teeth extracted at Karamanoğlu Mehmetbey University’s Ahmet Keleşoğlu Faculty of Dentistry for orthodontic treatment or due to periodontal problems, as well as naturally exfoliated deciduous teeth. In total, 112 teeth (56 deciduous and 56 permanent teeth) were used.

### Inclusion and exclusion criteria

We excluded carious and filled deciduous and permanent teeth.

#### Fluoride analysis, preparation of the enamel surface, and experimental groups

We analysed fluoride content using an ion-selective electrode (Thermo ScientificTM OrionTM Dual StarTM PH, ISE, mV, ORP, and Temperature Dual Channel Benchtop Meter, Japan) (Table 1). The final concentration of fluoride standards used in the calibration curve of the ORION Dual Star ionmeter device used as an ionmeter device is: 0.1 ppm-0.5 ppm-1 ppm-2 ppm. In the standards and samples, 50% TISAB II was used as a buffer. The method used is based on the determination of the electric potential in mV. The accuracy of the technique is also checked with the calibration curve and the verification solution prepared with NaF. The coefficient of variation is in the range of 0.005–0.007.

**Table 1:** Fluoride ion ratios of different drinking waters and tea brewed with these waters detected by ion selective electrode (mg/lt)

Group	Sample name	F (mg/L)
Group 1	Beysu water (Central Anatolia Region (Konya))	0,22
	Beysu water-brewed tea (Rize Tourist Tea (ÇAYKUR, Rize))	1,51
Group 2	Bulak water (Black Sea Region (Trabzon))	0,19
	Tea brewed with Bulak water (Rize Tourist Tea (ÇAYKUR, Rize))	0,95
Group 3	Hamidiye Water (Marmara Region (Istanbul))	0,22
	Tea brewed with Hamidiye water (Rize Tourist Tea (ÇAYKUR, Rize))	1,32
Group 4	Munzur water (Eastern Anatolia Region (Tunceli))	0,17
	Tea brewed with Munzur water (Rize Tourist Tea (ÇAYKUR, Rize))	1,31
Group 5	Nazlı water (Aegean Region (Aydın))	0,30
	Tea brewed with kind water (Rize Tourist Tea (ÇAYKUR, Rize))	1,43
Group 6	Sade life water (South Eastern Anatolia Region (Şanlıurfa))	0,22
	Tea brewed with plain life water (Rize Tourist Tea (ÇAYKUR, Rize))	1,08
Group 7	Ceysu water (Mediterranean Region (Antalya))	0,17
	Tea brewed with Ceysu water (Rize Tourist Tea (ÇAYKUR, Rize))	1,12

The teeth included in the study were stored in a 0.1% thymol solution until the procedure was performed. Cleaned of coarse debris, the enamel was separated under water cooling using an Isomet device at the Research Laboratory of the Faculty of Dentistry at Selcuk University, leaving it 1 mm shorter than the enamel–cementum junction (Isomet Low Speed Saw; Buehler Ltd, Lake Bluff, IL, USA). Following separation, the enamel samples were placed in separate containers and submerged for 15 min each in the water and tea. This duration mirrors the estimated consumption time for two litres of water and four cups of tea per day.

#### Tea brewing process

Tea brewing was conducted in a porcelain teapot with water heated to boiling point (100 °C). A porcelain teapot was used due to the potential for metal ion transfer to the tea at 100 °C if a metal teapot were employed [26]. Following the combination of boiling water and tea (5 g black tea and 160 mL water), the brew was allowed to steep in the porcelain teapot for a minimum of 10 min.

#### Tooth placement

Each water group had 14 teeth, consisting of 7 deciduous and 7 permanent teeth. The teeth were placed in different groups based on the water used: Group 1 utilized Beysu (Konya Suki Enerji Yatırım San. Tic. A.Ş., Konya, Turkey), Group 2 used Bulak (Bulak Su Meşrubat San. ve Tic. A.Ş., Trabzon, Turkey), Group 3 included Hamidiye (Hamidiye Spring Water, Istanbul Metropolitan Municipality, Istanbul, Turkey), Group 4 included Munzur (Munzur Water, Tunceli, Turkey), Group 5 incorporated Nazlı (ST Group, Aydın, Turkey), Group 6 contained Sade life (Sade Life Natural Spring Water, Şanlıurfa, Turkey), and Group 7 used Ceysu (Ceysu Natural Spring Water, Antalya, Turkey).

#### Tea treatment

The only brand of tea used was Çaykur Rize Tourist Tea (Çaykur (General Directorate of Tea Enterprises), Rize, Turkey). The teeth were treated with a fluoride gel (Polimo 1.23% APF, Imicryl) before immersion in the tea and water of Group 8, which used the Ceysu brand (Ceysu Doğal Kaynak Suyu, Antalya, Turkey), the water with the lowest fluoride content. The purpose of Group 8 was to observe the impact of the fluoride gel. Çaykur was selected for the study as it is the most preferred brand in Turkey [27]. This process was continued for 1 month.

#### Post-treatment procedure

When not under treatment, the enamel was stored in artificial saliva (Testonic Laboratories, Colin Kimya San. Tic. A.Ş. İstanbul, Turkey). The artificial saliva used is AFNOR artificial saliva solution used as electrolyte for electrochemical experiments. (pH: 8) Prepared according to Carter Brugigard AFNOR/NF French Association of Normalization 591 − 141 standard. Prior to scanning electron microscopy (SEM) examination, all samples were placed in containers containing 1 mol/L KOH for 8 h at room temperature. This step was necessary to remove surface debris and CaF2-like globules [28]. The fluoride content in the enamel structure of the teeth was evaluated using an SEM EDX device before and after the experiment (Images 1–10).

**Fig. 1:**
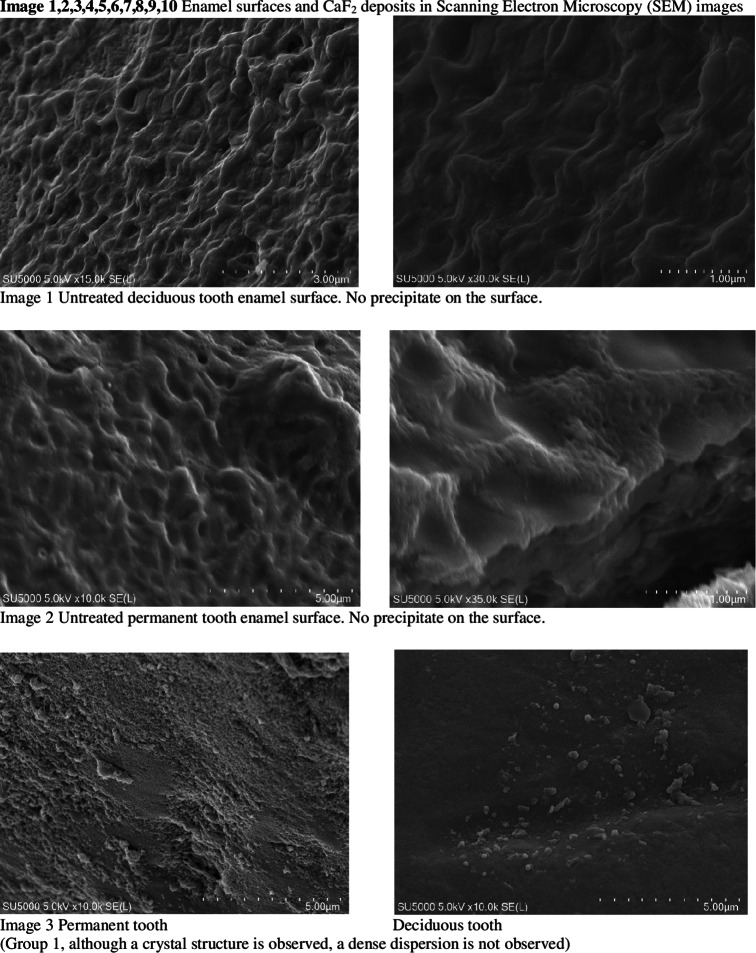

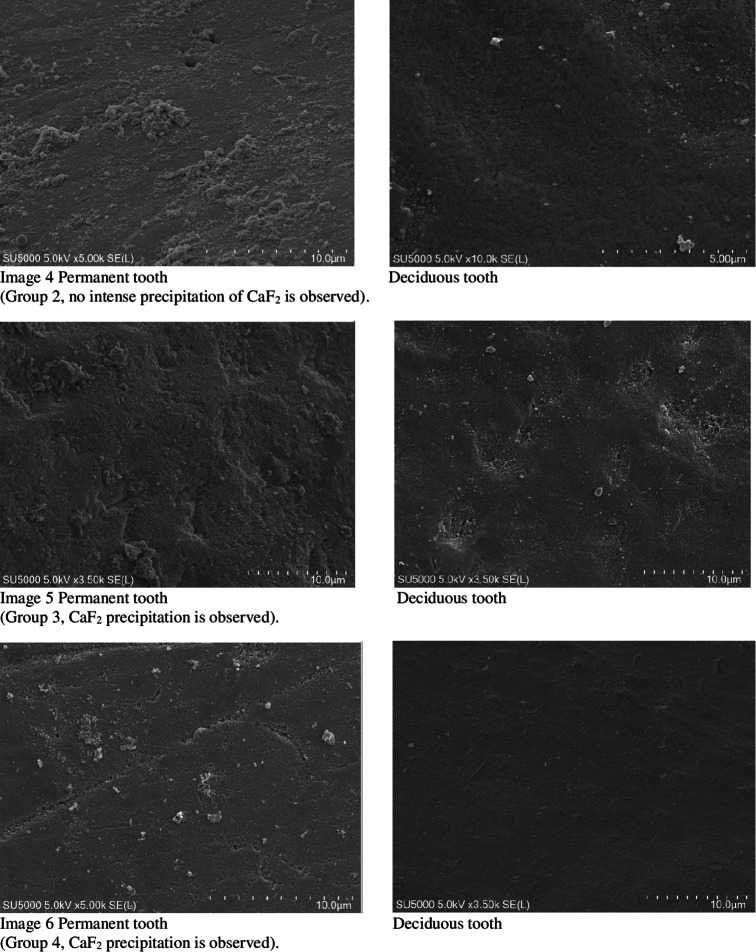

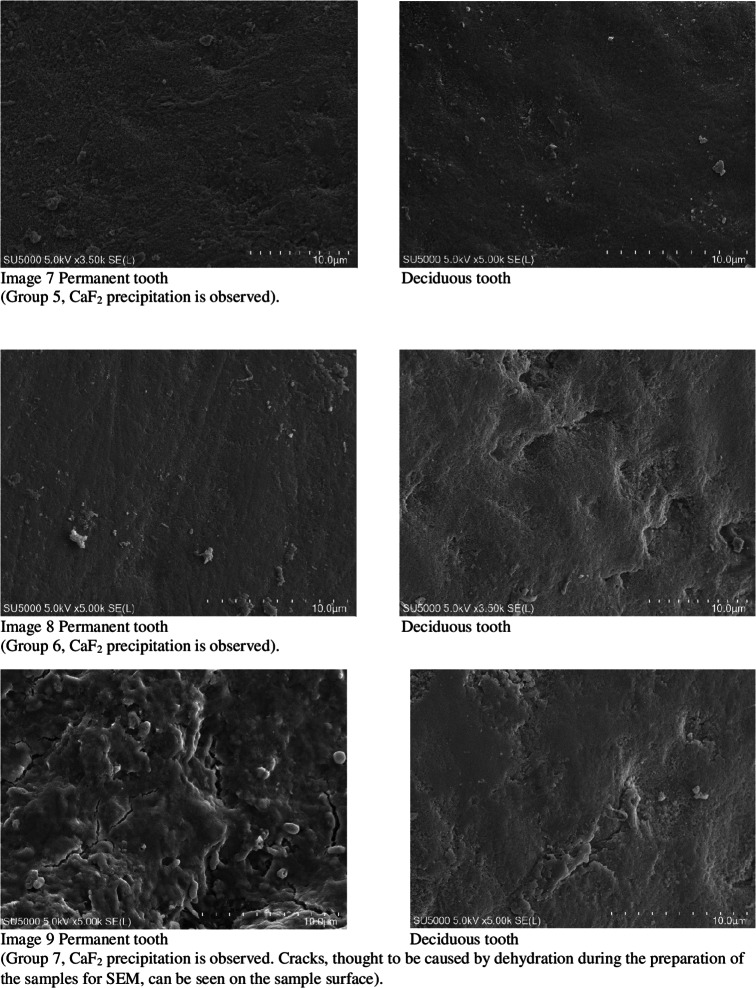

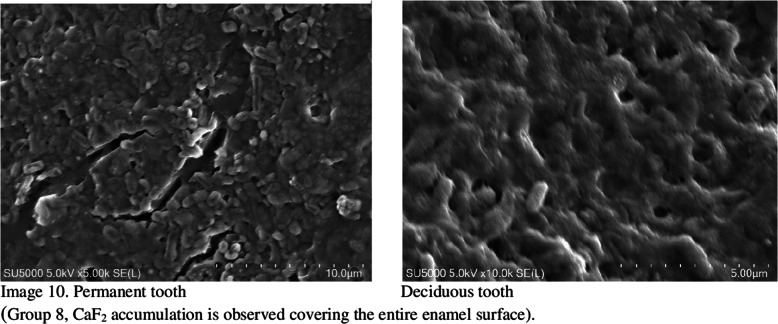
Images 1–10 Enamel surfaces and CaF2, deposits in Scanning Electron Microscopy (SEM) images There are 10 images in total, but only three are included here. 7 pictures are missing. On the following pages of the Tiff file are the continuation of the pictures. At the same time, tiff files were divided into pages and uploaded as additional material.

### Statistical analysis

For the statistical evaluation, an initial normality test was conducted on the data, which showed that the data were normally distributed. Consequently, we decided to use a parametric test for further analysis. The impact of two factors on the detection of fluoride in teeth via SEM EDX was examined. The first factor was the distinction between deciduous and permanent teeth, and the second factor was the differences among various water brands. The influence of these two factors was assessed using a two-way ANOVA test utilizing SPSS v.27 software (p = 0.05). A *post hoc* Tukey test was subsequently conducted to identify the difference between water brands.

## Results

The fluoride ion ratios (mg/lt) of the different drinking waters detected by the ion selective electrode and the tea brewed with these waters are given in Table 1.

The fluoride content of enamel of permanent teeth was statistically significantly lower than that in deciduous teeth. Furthermore, regarding the different water brands, Nazlı Water had the highest levels while Ceysu Water had the lowest (Table 2).

**Table 2:** Fluoride amounts of teeth kept in drinking water and tea; (Atomic %) (Average±SS)

Water Teeth	Beysu	Bulak	Hamidiye	Munzur	Nazlı	Sade Life	Ceysu	Fluoride + Ceysu
Permanent teeth	22,98±0,42^Aa*^	14,95±0,45^Ab^	20,63±1,47^Ac^	11,06±0,53^Ad^	26,06±0,70^Ae^	19,18±0,32^Af^	9,21±0,92^Ag^	65,87±0,58^Ah^
Deciduous teeth	24,17±0,47^Ba^	16,13±0,30^Bb^	21,50±1,19^Bc^	12,00±0,67^Bd^	26,67±0,75^Be^	19,97±0,16^Bf^	9,43±0,88^Bg^	75,33±0,63^Bh^

* In the table, capital letters were used for statistical comparison of deciduous and permanent teeth and lower case letters were used for comparison of water brands. (p < 0.05)

SEM images did not detect fluoride on untreated enamel surfaces. This may be because the enamel layer is primarily composed of hydroxy-apatite molecules, which have a crystallized calcium phosphate structure. Fluoride can reduce dental mineral solubility by substituting the hydroxyl groups and lowering the carbonate content. Consequently, fluoride aids mineral precipitation or redeposition by reducing the solubility products of precipitated calcium phosphates [29].

## Discussion

Given that not all individuals in society can access dental services, systemic fluoride applications are of great importance. In this regard, Lee et al. found that increased access to fluoridated water for a population was linked to a decrease in caries-related visits [30]. Slade et al. found that water fluoridation is effective for preventing caries, particularly in deciduous teeth [31]. Our results are in agreement; we found that fluoride precipitation in enamel immersed in drinking water and tea was statistically significantly higher in deciduous teeth than in permanent teeth. Likewise, Meyer et al. found that exposure to optimally fluoridated water effectively prevents dental caries [32]. Nor et al. discovered that a change in fluoride level from 0.7 ppm to 0.5 ppm reduced fluorosis while retaining its caries preventive effect [33].

Several studies that have assessed fluoride levels in drinking water have been conducted in our country over different years. Dursun et al. measured fluoride concentrations in water samples taken from 50 drinking water wells in the city centre of Konya and found that levels in all samples were less than 0.8 mg/L, closely aligning with the minimum required value according to Turkish Standards for Drinking Water (TS 266) [34, 35]. Şener et al. investigated the fluoride levels of three different spring waters in Konya province and reported levels between 0.006 and 0.124 ppm [36]. Boyraz explored the effect of household water treatment devices on drinking water quality and discovered that the fluoride, calcium, and magnesium values were significantly lower than those in pure tapwater [37]. In addition, Ermiş et al. investigated the severity of dental caries and fluorosis in children living in areas with low and high fluoride water in Turkey, and discovered that increased fluoride levels were associated with a higher prevalence of dental fluorosis but had no impact on caries experience in children with poor oral hygiene [38]. By contrast, Sezgin et al. evaluated all children aged 7–13 years in the village of Hanlıyenice, Turkey, where the drinking water had a fluoride level 2.5 times the optimal amount and found that high fluoride exposure increased fluorosis levels but significantly reduced dental caries [39]. In our study, the fluoride levels in bottled drinking water ranged between 0.17 and 0.30 mg/L, lower than the level recommended by the WHO for drinking water.

The aforementioned studies evaluated the caries rate in the population based on the fluoride level of drinking water but did not consider the impact of fluoride content of daily consumed beverages, such as tea and water, on tooth enamel. Drinking water with high fluoride levels, and tea brewed with this water, resulted in more significant fluoride deposition on tooth enamel.

Fluoride, an essential element in healthy tooth enamel, is present at 0.016% [40]. We speculate that the absence of fluoride in the tooth enamel evaluated in our study may be due to the fact that the teeth were kept in KOH before the application. The WHO has indicated that fluoride levels of 0.5–1.2 ppm in drinking water contribute to reducing caries prevalence [41]. In a study that examined the fluoride level of drinking water in İzmir province, it was found that levels are below the optimal value [42]. Ataç et al. examined the fluoride levels in 13 different brands of bottled water and detected levels below 0.3 ppm in 10 of the samples and levels around 1 ppm in only 3 of the samples [43]. In our study, the fluoride level in bottled drinking water was below the optimum value, and the highest concentrations (0.30 mg/L) were found in Nazlı water from the Aegean Region.

Rajkovic et al. reported that detecting fluoride content in drinking water and tea infusions using ion-selective electrodes was simple, inexpensive, and reliable [44]. Gilijanovic et al. analysed fluoride levels of 43 tea infusions, including mint (*Mellissa officinalis*), green tea (*Camellia sinensis*), and pomegranate (*Punica granatum*) sold in supermarkets and local markets in the Split region of Croatia. The concentrations in bulk samples (0.008 ± 0.003 mg/L) were lower than in tea bags and bottled tea (0.191 ± 0.116 mg/L) [45]. In our study, fluoride analysis was performed using an ion-selective electrode, and the content in bottled drinking water was lower than in tea brewed with the same water.

Amanlou et al. analysed fluoride levels in bottled water from 18 different brands in Iran. Six samples were taken separately from each brand and had 0.202 ± 0.00152 mg/L fluoride, below the accepted limits. These findings indicate that bottled water contains lower fluoride levels than tap water, which suggests that the risk for fluorosis could be reduced by choosing bottled water. However, because people drinking bottled water would have a low intake of fluoride, there might be an increased risk for dental caries, and fluoride supplementation is recommended for people who prefer bottled water [46].

In our study, the fluoride ratio was considerably high in teeth pretreated with fluoride before exposure to water and tea. In this context, we evaluated the effects of regional drinking water and tea, commonly consumed in Turkey, on dental caries in rural populations with limited access to dental services. This was done to underscore the importance and need for topical fluoride applications.

We kept samples in artificial saliva. However, given the constant flow of saliva in the human mouth, the cleansing effect of saliva cannot be overlooked. Another limitation to consider is the staining effect of tea on tooth enamel [47, 48].

## Conclusion

We evaluated the effects of fluoride content in tea and water on tooth enamel. The amount of fluoride was lower in bottled drinking water than in brewed tea. Particularly for individuals living in rural areas with limited access to dental care, fluoride intake through daily beverage consumption is crucial. On the other hand, the fluoride ratio in tooth enamel was quite high in teeth exposed to topical fluoride; thus, ideally, fluoride intake should be supplemented with topical fluoride application. Furthermore, our results suggest that easier access to black tea and fluoridated water in daily life could be associated with a decrease in caries-related visits to the dentist.

## Data Availability

The datasets used and analysed during the current study are available from the corresponding author on reasonable request.
